# StraPep: a structure database of bioactive peptides

**DOI:** 10.1093/database/bay038

**Published:** 2018-04-16

**Authors:** Jian Wang, Tailang Yin, Xuwen Xiao, Dan He, Zhidong Xue, Xinnong Jiang, Yan Wang

**Affiliations:** 1 Key Laboratory of Molecular Biophysics of the Ministry of Education, College of Life Science and Technology, Huazhong University of Science and Technology, Wuhan, Hubei 430074, China; 2 Reproductive Medicine Center, Renmin Hospital of Wuhan University, Wuhan, Hubei 430060, China; 3 School of Software Engineering, Huazhong University of Science and Technology, Wuhan, Hubei 430074, China; 4 Department of Neurology, The First Affiliated Hospital, Sun Yat-sen University, Guangzhou, Guangdong 510080, China

## Abstract

Bioactive peptides, with a variety of biological activities and wide distribution in nature, have attracted great research interest in biological and medical fields, especially in pharmaceutical industry. The structural information of bioactive peptide is important for the development of peptide-based drugs. Many databases have been developed cataloguing bioactive peptides. However, to our knowledge, database dedicated to collect all the bioactive peptides with known structure is not available yet. Thus, we developed StraPep, a structure database of bioactive peptides. StraPep holds 3791 bioactive peptide structures, which belong to 1312 unique bioactive peptide sequences. About 905 out of 1312 (68%) bioactive peptides in StraPep contain disulfide bonds, which is significantly higher than that (21%) of PDB. Interestingly, 150 out of 616 (24%) bioactive peptides with three or more disulfide bonds form a structural motif known as cystine knot, which confers considerable structural stability on proteins and is an attractive scaffold for drug design. Detailed information of each peptide, including the experimental structure, the location of disulfide bonds, secondary structure, classification, post-translational modification and so on, has been provided. A wide range of user-friendly tools, such as browsing, sequence and structure-based searching and so on, has been incorporated into StraPep. We hope that this database will be helpful for the research community.

**Database URL**: http://isyslab.info/StraPep

## Introduction

Bioactive peptides are peptides with hormone or drug like activity, and most of them are generated by proteolytic cleavage of large prepropeptides. As important signaling molecules, bioactive peptides interact with specific cell surface receptors, cytokines or other signaling proteins and regulate a variety of biological and physiological responses (1). For example, neurotransmitter enables communication between synapses; antimicrobial peptides are the first line of defense against pathogen infections; and venom peptides can modulate the activity of ion channels. Due to the diverse biological activities and universal distribution in nature, bioactive peptides have attracted great research interest in biological, medical and industrial fields, especially in pharmaceutical industry (2).

Currently, >60 peptide drugs have been marketed and approximately 140 peptides are under evaluation by clinical trials, with >500 peptides in the preclinical development (3–5). The strengths of bioactive peptides in their use as therapeutics include: (i) good efficacy, safety and tolerability; (ii) high selectivity and potency; and (iii) predictable metabolism. However, due to the physiochemical instability, tendency for aggregation and short plasma half-life, natural peptides are often not directly suitable for use as convenient therapeutics. Rational design of peptide therapeutics, starting with a known crystal structure of the peptide, can mitigate the weaknesses of natural peptides by building the structure-activity relation (SAR) that helps to identify essential amino acids and sites for possible substitution. Modifications introduced into a given peptide via rational design can improve its physiochemical properties (4). For example, glucagon-like peptide-1 (GLP-1), a 30 or 31 residue peptide hormone (^7^HAEGTFTSDVSSYLEGQAA***K***^26^EFIAWLV***K***^34^GRG^37^, the last G residue may not be present) composed of two α-helices (underlined sequences) separated by a linker region, is rapidly degraded by dipeptidyl peptidase-4 (DPP-4) *in vivo*, resulting in a plasma half-life of ∼2 min. Liraglutide (Victoza, Saxenda) is an analog of GLP-1 generated by acylation of Lys^26^ and replacing Lys^34^ with Arg. Such modification based on SAR of GLP-1 retains the potent of GLP-1 but promotes self-association and noncovalent binding to the fatty acid binding sites of plasma albumin, resulting in reduced or no affinity for DPP-4. Liraglutide has a half-life of 13 h and is used as a long-acting GLP-1 receptor agonist to stimulate insulin secretion. Liraglutide has been marketed for the treatment of type 2 diabetes mellitus since 2009 (6).

In the past, many databases have been developed to maintain different kinds of peptides (Table 1). The top four databases in Table 1 focus on candidate peptide discovery. BactPepDB (7) predicts peptides from an exhaustive survey of complete prokaryote genomes. BAGEL2 (8), Effective (9) and C-PAmP (10) are databases of predicted bacteriocins, secreted bacterial proteins and antimicrobial peptides of plant origin, respectively. The remaining databases in Table 1 collect peptides from public databases and/or literature and most of them are designed for one specific type of bioactive peptides. For example, Amper (11), APD (12), Bactibase (13), CAMP (14), DAMPD (15) and YADAMP (16) hold antimicrobial peptides; Quorumpeps (17) is developed for quorum sensing peptides; CPPsite (18) and CPPsite 2.0 (19) are established to provide comprehensive information of cell-penetrating peptides; ArachnoServer (20) and ConoServer (21) focus on venom toxin peptides; DADP (22) holds anuran defense peptides; NORINE (23) is a database of nonribosomal peptides; databases www.neuropeptides.nl (24), Neuropedia (25) and NeuroPep (26) are dedicated to neuropeptides. SATPdb (27), which integrates 22 public peptide databases, is a database of structurally annotated therapeutic peptides and has 19 192 peptide sequences with length between 2 and 50 amino acids, among which only 644 peptides have experimental structures. So far, only several databases are developed to maintain general known bioactive peptides. PeptideDB (2) collects bioactive peptide sequences and corresponding precursor proteins of metazoan species from UniprotKB. PepBank (28) is consisted of almost 20 000 peptides with length ≤20, which are mainly extracted from text mining of MEDLINE abstracts. EROP-Moscow (29) contains natural oligopeptides that are collected directly from publications in scientific journals. However, to the best of our knowledge, there is no database focusing on all the bioactive peptides with known structure. To construct a structure database of bioactive peptide, we focused on collecting as many bioactive peptides with known structures as possible in this study. This database will be a valuable complement to the current peptide databases.

**Table 1.** The bioactive peptide databases

Name	Data source	Peptide type	Structure annotation	References
BactPepDB	Prediction	Bacterial peptide	Prediction	7
BAGEL2	Prediction	Antimicrobial peptide	No	8
Effective	Prediction	Secreted bacterial protein	No	9
C-PAmP	Prediction	Antimicrobial peptide	Prediction	10
Amper	Public database	Antimicrobial peptide	No	11
APD	Public database and literature	Antimicrobial peptide	PDB	12
Bactibase	Public database and literature	Antimicrobial peptide	PDB	13
CAMP	Public database	Antimicrobial peptide	PDB	14
DAMPD	Public database	Antimicrobial peptide	No	15
YADAMP	Public database	Antimicrobial peptide	No	16
Quorumpeps	Literature	Quorum Sensing peptide	PDB	17
CPPsite	Literature	Cell-penetrating peptide	PDB and prediction	18
CPPsite 2.0	Literature	Cell-penetrating peptide	PDB and prediction	19
ArachnoServer	Public database	Venom toxin peptide	PDB	20
ConoServer	Public database&Literature	Venom toxin peptide	PDB	21
DADP	Public database and literature	Anuran defense peptide	No	22
NORINE	Literature	Nonribosomal peptide	No	23
www.neuropeptides.nl	Public database	Neuropeptide	No	24
Neuropedia	Public database and literature	Neuropeptide	No	25
NeuroPep	Public database and literature	Neuropeptide	PDB	26
SATPdb	Public database	Therapeutic peptide	PDB	27
PeptideDB	Public database	Bioactive peptide	No	2
PepBank	Public database and literature	Peptide	No	28
EROP-Moscow	Literature	Oligopeptide	No	29

## Materials and methods

### Data collection and compilation

**Figure 1.**
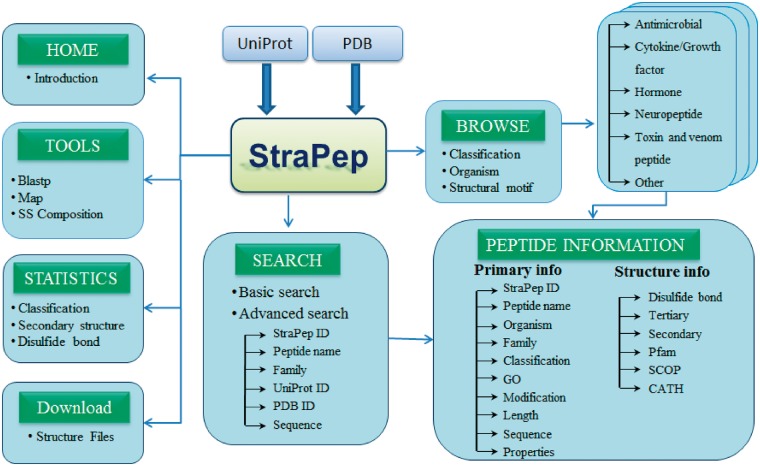
The overall architecture of StraPep.

StraPep is a bioactive peptide structure database. The architecture of StraPep is shown in Figure 1. The data were collected from UniProtKB (30) and PDB (31). Similar to the schemes used by PeptideDB, the bioactive peptide entries were retrieved from UniProtKB when it was annotated as bioactive peptide in the ‘Features’ line, or its protein name contained peptide as keyword, or it was annotated with peptide keywords in the ‘Keywords’ line. The peptide keywords include amphibian defense peptide, antimicrobial, cytokine, growth factor, hormone, neuropeptide, neurotransmitter, opioid peptide, pheromone, toxin and vasoactive. After excluding non-peptide entries by manual checking, 3293 structures belonging to 1019 unique bioactive peptide sequences were extracted. To retrieve bioactive peptide without annotation in UniProtKB, we further checked PDB entries classified as toxin, hormone, antibiotic, antimicrobial and so on, and 293 more peptides were collected.

### StraPep database and web interface

**Figure 2.**
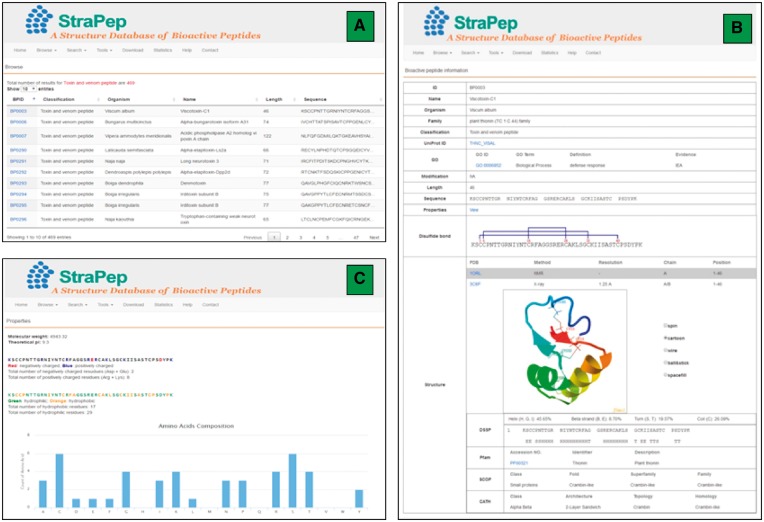
An overview of the user interface of the web interface of StraPep. (**A**) The browse output of toxin and venom peptides. (**B**) Entry BP0003 as an example of toxin and venom peptides. ID: a unique code to identify each database entry. Organism: the scientific name of the organism producing the peptide. Classification: the classification of the peptide, which was based on the function annotation of the peptide in UniProt or PDB. Modification: the type of posttranslational modification and the position of each modified residue in the sequence. Structure: the three-dimensional structure of the peptide, the secondary structure information derived from DSSP, and the domain information of the peptide in Pfam, SCOP and CATH (if available) are shown. (**C**) The properties page of the entry BP0003. the frequency of each common amino acids, the number of charged, hydrophilic and hydrophobic amino acids, the isoelectric points and molecular weight of each peptide were computed.

The StraPep database was constructed with Apache Server 7 with MySQL Community Server 5.6. HTML, CSS, Javascript and Bootstrap framework were used to build the front-end, and PHP was used to implement the web services. Figure 2 illustrates an overview of the user interface of StraPep database.

### Data retrieval

To facilitate users in retrieving the data from StraPep, we integrated powerful browsing and searching tools into the website. Users can browse the peptide entries by four different major categories including organism, classification, disulfide bond(s) and the cystine knot motif. There are 452 organisms in the current database, which is presented in alphabetical order. Based on the curated information of functions, peptides in StraPep have been grouped into six major functional categories including antimicrobial peptide, toxin and venom peptide, cytokine and growth factor, hormone, neuropeptide and others. Users can also browse the peptides based on the number of disulfide bonds, which range from 0 to 8, and cystine knot motifs including inhibitor cystine knot (ICK), cyclic cystine knot (CCK) and growth factor cystine knot (GFCK). The browse output has the option for sorting the data by clicking the column title. Furthermore, users can query the database by two types of search tools: quick search and advanced search. Quick search enables users to search the database by the following fields: ID, name, UniProt ID, PDB ID and sequence. Advanced search allows a combination of several fields via using logical conditions like AND/OR.

### Integration of web tools

Various web-based tools were integrated into StraPep to facilitate the search and analysis of bioactive peptide with known structure. A brief description of these tools is as follows.

*Blastp*: To find whether there are solved peptide structures in StraPep that are similar to a user-provided sequence, the Blastp search tool has been incorporated into the website (32). It allows users to submit the sequence in FASTA format and choose the user-defined parameters including E-value cutoff and the substitution matrix for sequence alignment. The output is shown in the standard Blastp output, which includes the matching sequences, Blastp score and E-value.

*Map*: Given the sequence of a peptide precursor, one may need to find all possible processed peptides with known 3 D structure in the database. To facilitate this task, we have developed the Map tool, which finds all peptides in the database that exactly match to a substring in the query sequence.

*SS composition*: This interface is designed for retrieving peptides according to their secondary structure composition. Secondary structures were classified into helix (H, G, I), beta strand (E, B), turn (T, S) and coil (C) based on the DSSP state. It allows users to obtain peptides with the preset composition of the four types of secondary structure states.

## Results and discussion

After manual checking and removal of redundancy, the current release of the database (version 1.0) holds 1312 unique bioactive peptides including 464 toxins and venom peptides, 404 antimicrobial peptides, 217 cytokines and growth factors, 141 hormones, 39 neuropeptides and 47 others. Each unique peptide has at least one solved structure in PDB, and 513 peptides have two or more known structures. Consequently, there are 3791 bioactive peptide structures determined by NMR and X-ray in total, including 885, 833, 901, 860, 60, and 252 structures of toxin and venom peptide, antimicrobial peptide, cytokine and growth factor, hormone, neuropeptide and others, respectively.

### Secondary structure and peptide length distribution

**Figure 3.**
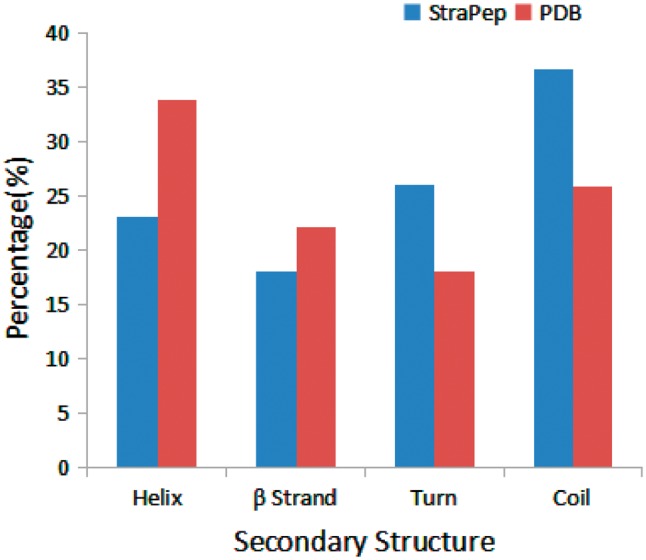
Comparison of secondary structure compositions of StraPep and PDB.

**Figure 4.**
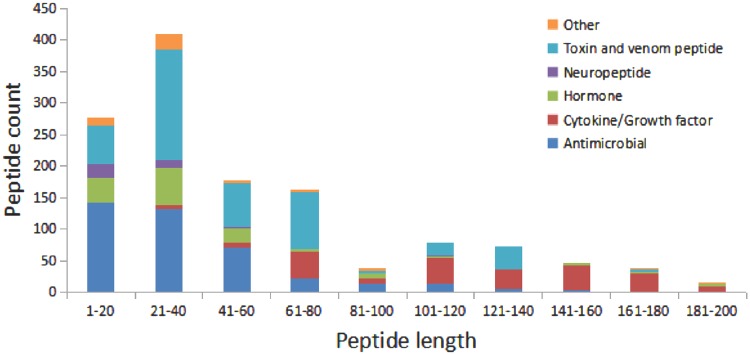
The length distribution of the different kinds of peptides in Strapep.

We have compared the secondary structure composition of proteins collected in PDB and peptides in StraPep. It is found that the peptides in StraPep contain less regular secondary structure (helix and beta strand) but more non-regular structure (turn and coil), and the percentage of turn and coil are 1.44 and 1.41 times of that in PDB, respectively (Figure 3), which is conceivable since it is difficult for a short peptide to maintain a regular secondary structure. The length distribution of the different kinds of peptides in Strapep has also been computed and is shown in Figure 4. The length of most of the peptides (81%) in Strapep is <100; 60% peptides with length >100 are cytokines and growth factors. The shortest peptide with known 3 D structure is five amino-acids long.

### Structural motif analysis of peptides in StraPep

**Figure 5.**
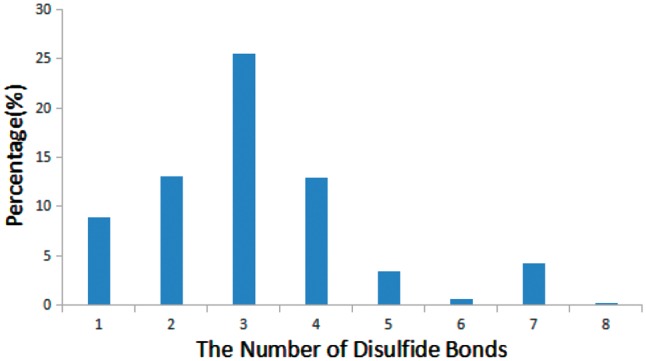
The percentage of peptides with various number of disulfide bonds in StraPep.

Disulfide bonds are common in many bioactive peptides and are critical for the structure and function of peptides (33). We found that 68% of bioactive peptides (905 out of 1312) in StraPep contain disulfide bonds, while only 21% of proteins in PDB contain disulfide bonds. Moreover, the percentage of disulfide bond-containing peptides varies greatly in different categories in StraPep, with 92% in toxin and venom peptides, followed by 80% in cytokine/growth factor, 51% in hormone and 48% in antimicrobial. Peptides have up to eight disulfide bonds in the current version of StraPep. Figure 5 illustrates the percentage of peptides with various number of disulfide bonds (1–8) in StraPep. Of note, >26% peptides contain three disulfide bonds, followed by peptides containing two and four disulfide bonds. Interestingly, 57 (4.3%) peptides that have 7 disulfide bonds contain 14 Cys residues in total and 2 (0.1%) peptides with 8 disulfide bonds contain 16 Cys residues in total.

Peptides with three or more disulfide bonds may form a structural motif known as cystine knot, which is characterized by a loop formed by two disulfide bridges through which a third disulfide bond passes. It confers considerable structural stability on peptides and is an ideal framework for the development of potential therapeutic or diagnostic agents (34). In StraPep, 616 peptides contain three or more disulfide bonds, and 150 (24%) of them form cystine knot, among which 88 are ICKs, 21 are CCKs and 41 are GFCKs. It has been reported that the ICK motif has a variety of diagnostic applications (35). For example, chlorotoxin (BP0360), a scorpion venom peptide, can cross blood–brain barriers and specifically bind to malignant glioma. Synthetic version of chlorotoxin has been evaluated in phase II human clinical trials under the name TM-601 to treat and image malignant glioma (36). Moreover, fluorescent dye conjugated chlorotoxin can be used as a ‘tumor paint’ to delineate the margins of glioma and hence facilitate their surgical removal (37).

### Comparison to other existing peptide databases

Strapep is a database which focusing on collecting as many bioactive peptides with known structures as possible, therefore we compare it with databases which collect data from public databases and/or literature and hold more than one kind of bioactive peptide(the last four databases in Table 1). SATPdb (27) included 644 therapeutically important peptides with known structure, whereas StraPep collected more types of bioactive peptides and two times more peptides with known structure. As seen from Table 1, PeptideDB (2), PepBank (28) and EROP-Moscow (29) did not provide any 3 D structure annotation. In summary, StraPep is a valuable complement to the other existing peptide databases.

## Summary and future perspectives

StraPep is a public resource of all bioactive peptides with known structure, which holds 3791 bioactive peptide structures originating from 1312 unique bioactive peptides with at least one solved structure in PDB. The database provides not only a user-friendly interface coupled with powerful browsing, searching and analysis tools to facilitate the search and analysis of bioactive peptide with known structure but also a benchmark dataset for the development of peptide structure prediction tools. In addition, our team will update the database every 6 months. Therefore, we think the StraPep database will be a useful resource for the community.
